# Saikosaponin a Mediates the Anticonvulsant Properties in the HNC Models of AE and SE by Inhibiting NMDA Receptor Current and Persistent Sodium Current

**DOI:** 10.1371/journal.pone.0050694

**Published:** 2012-11-29

**Authors:** Yun-Hong Yu, Wei Xie, Yong Bao, Hui-Ming Li, San-Jue Hu, Jun-Ling Xing

**Affiliations:** 1 Department of Traditional Chinese Medicine, Nanfang Hospital, Southern Medical University, Guang Zhou, People’s Republic of China; 2 School of Traditional Chinese Medicine, Southern Medical University, Guang Zhou, People’s Republic of China; 3 Institute of Neuroscience, Fourth Military Medical University, Xi’an, People’s Republic of China; 4 Department of Neurology, Traditional Chinese Hospital of Lu’an, Lu’an, People’s Republic of China; Universidade Federal do Rio de Janeiro, Brazil

## Abstract

Epilepsy is one of the most common neurological disorders, yet its treatment remains unsatisfactory. Saikosaponin a (SSa), a triterpene saponin derived from *Bupleurum chinensis* DC., has been demonstrated to have significant antiepileptic activity in a variety of epilepsy models in vivo. However, the electrophysiological activities and mechanisms of the antiepileptic properties of SSa remain unclear. In this study, whole-cell current-clamp recordings were used to evaluate the anticonvulsant activities of SSa in the hippocampal neuronal culture (HNC) models of acquired epilepsy (AE) and status epilepticus (SE). Whole-cell voltage-clamp recordings were used to evaluate the modulation effects of SSa on NMDA-evoked current and sodium currents in cultured hippocampal neurons. We found that SSa effectively terminated spontaneous recurrent epileptiform discharges (SREDs) in the HNC model of AE and continuous epileptiform high-frequency bursts (SE) in the HNC model of SE, in a concentration-dependent manner with an *IC_50_* of 0.42 µM and 0.62 µM, respectively. Furthermore, SSa significantly reduced the peak amplitude of NMDA-evoked current and the peak current amplitude of *I_NaP_*. These results suggest for the first time that the inhibitions of NMDA receptor current and *I_NaP_* may be the underlying mechanisms of SSa’s anticonvulsant properties, including the suppression of SREDs and SE in the HNC models of AE and SE. In addition, effectively abolishing the refractory SE implies that SSa may be a potential anticonvulsant candidate for the clinical treatment of epilepsy.

## Introduction

Epilepsy is one of the most common neurological disorders with an incidence of approximately 0.3–0.5% [1]. It is characterized by the occurrence of spontaneous, recurrent, unprovoked seizure discharges resulting from abnormal, disordered, spontaneous or synchronized, and high-frequency firing of neuronal populations in the central nervous system. Despite the fact that there are a large number of available antiepileptic drugs (AEDs) in the market, the treatment of epilepsy remains unsatisfactory in terms of adverse effects and susceptibility to clinically important drug-drug interactions [2]. Approximately 40% of the patients are still refractory to treatment with conventional AEDs [3]. Thus, there is a growing demand for developing newer medications to treat epilepsy.

The hippocampal neuronal culture (HNC) model of acquired epilepsy (AE) is a well-established model that exhibits spontaneous recurrent epileptiform discharges (SREDs) for the life of the cultured hippocampal neurons [4] and has been routinely used to characterize biochemical, electrophysiological and molecular mechanisms underlying epilepsy in an in vitro setting [5]. The HNC model of status epilepticus (SE) that displays continuous epileptiform high-frequency bursts (SE) is often resistant to conventional anticonvulsant treatments and has been widely used to evaluate the effects of SE on neuronal cell physiology and molecular changes [6]. The two HNC models of AE and SE are widely used for pharmacological study of epilepsy.

**Figure 1:**
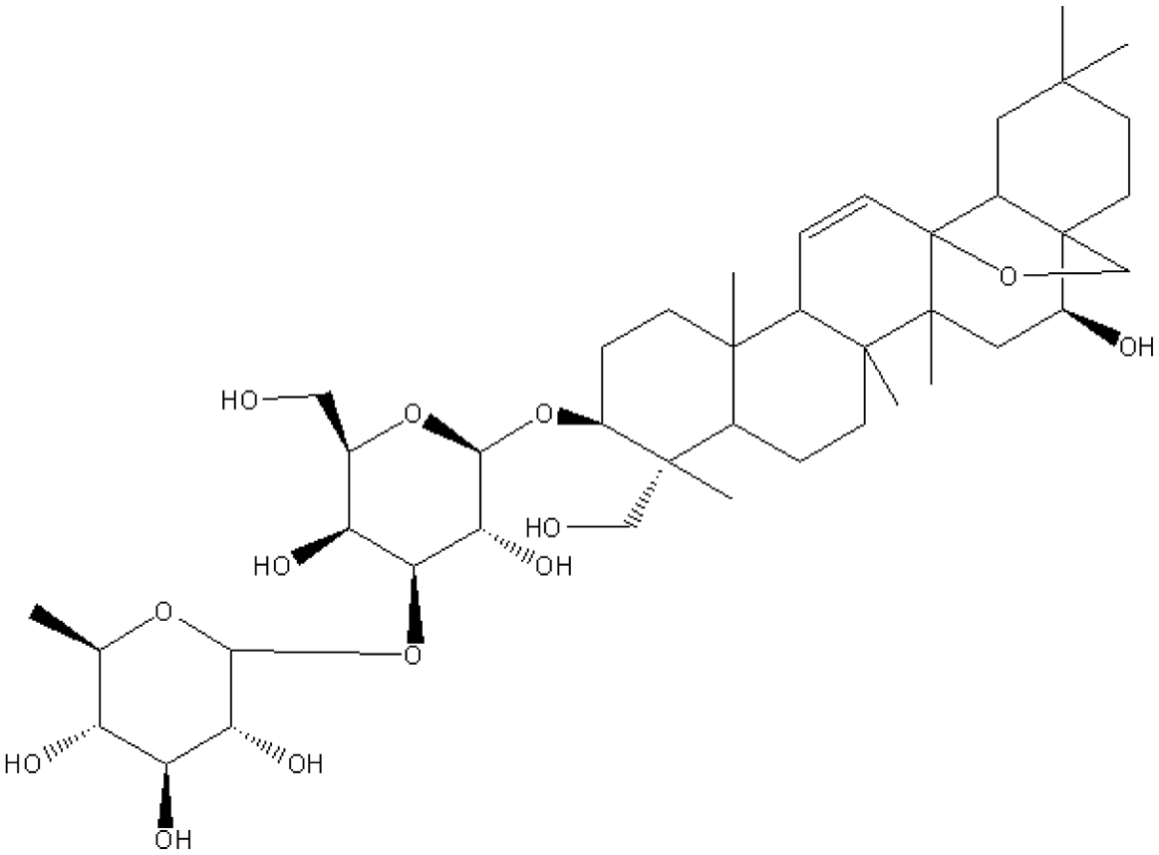
Chemical structure of Saikosaponin a (SSa). SSa is a triterpene saponin derived from *Bupleurum falcatum L*. It has variety of pharmacological activities, including anti-inﬂammatory, immunomodulatory, anti-bacterial, antidepression and anticonvulsant activities.

The exhibitions of SREDs and SE in low-Mg^2+^ solution in cultured hippocampal neurons are because of the elimination of Mg^2+^, a blocker of the ionotropic NMDA receptor channel, which is normally re-established after neuronal depolarization [7]. Several studies suggested that exposure to a low-Mg^2+^ solution, that reduced the blocking of NMDA receptor channel, produced repetitive depolarizations, increased transmitter release presynaptically, enhanced NMDA receptor-mediated excitability postsynaptically and enhanced the extent of neuronal interconnectivity elevating network excitability in vitro SREDs and SE [6], [8]. NMDA receptor current may change in the HNC models of AE and SE. On the other hands, sodium channels play a fundamental role in the reliable transmission of action potentials and in the generation of repetitive (burst) firing [9]. The abnormal function of sodium currents, including the persistent sodium current (*I_NaP_*) [10] and the transient sodium current (*I_Nat_*) [11], increases the excitability of neurons and contributes to the generation and spread of abnormal discharges and epileptic seizures. Moreover, the inhibition of sodium currents is commonly accepted to represent a prominent antiepileptic mechanism [12], such as the inhibition of *I_NaP_* [13].

Saikosaponin a (SSa) is a triterpene saponin (chemical structure shown in Fig. 1) derived from the medicinal plant, *Bupleurum chinensis* DC. (Umbelliferae) [14]. In laboratory studies, it was reported that SSa had variety of pharmacological benefits, including anti-inﬂammatory, immunomodulatory, and anti-bacterial activities [15]. In addition, several studies showed that SSa increased the levels of monoamine neurotransmitters, including homovanillic acid, noradrenaline, hydroxyphenyl ethylamine and serotonin, in the brain of “depressed” rats [16]. In recent years, we have found that SSa demonstrated antiepileptic activity in a variety of in vivo seizure models including the pentylenetetrazole kindling model, the maximal electroshock (MES)-induced seizure model as well as the Li-Pilocarpine induced refractory epilepsy [17], [18], [19]. SSa inhibited expression of glial fibrillary acidic protein and activation of rat hippocampal astrocytes induced by glutamate [20]. It also decreased [Ca^2+^] and decreased the secretion of IL-6 from glutamate-activated rat hippocampal astrocytes [21]. However, the exact anticonvulsant mechanism(s) of SSa on neurons and the electrophysiological mechanisms of the anticonvulsant activities is unknown.

**Figure 2:**
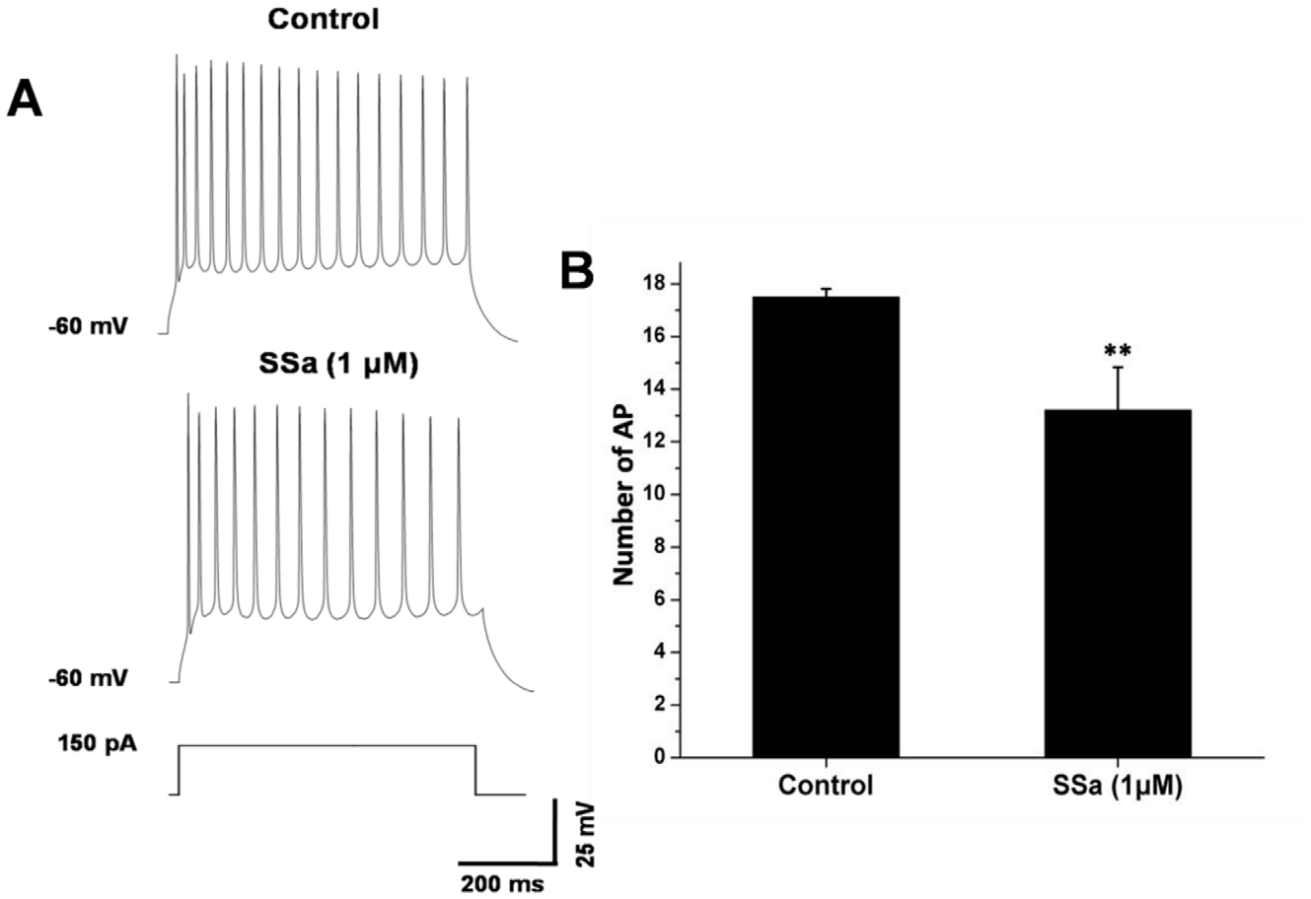
SSa decreases the number of action potentials of cultured hippocampal neurons induced by depolarizing current injection. (A) Whole-cell current-clamp recordings obtained (at 32–33°C, at a holding potential of −60 mV) from a cultured hippocampal neuron in response to depolarizing current injection (150 pA, 600 ms, bottom) in control condition (with vehicle, top) and in the presence of SSa (1 µM, middle). (B) The comparison of average number of action potentials elicited in hippocampal neurons before (control) and after applying SSa (1 µM; n = 10; paired t-test; **P<0.01).

**Table 1:** Effects of SSa on membrane properties of cultured hippocampal neurons.

	Resting membranepotential (mV)	Input resistance (MΩ)
Control	−61.2±0.5	165.3±12.1
SSa	−60.9±0.6	168.5±8.5

SSa (1 µM) did not produce any significant effect on the resting membrane potential and the input resistance of cultured hippocampal neurons (n = 10; paired t-test; *P*>0.05 vs. control).

The current study was initiated to investigate the anticonvulsant activities of SSa and its underlying anticonvulsant mechanisms. Using whole-cell current-clamp recordings, we found that SSa effectively terminated SREDs and SE in the HNC models of AE and SE. We further evaluated the modulation effects of SSa on NMDA-evoked current and sodium currents, including *I_NaP_* and *I_Nat_*, in cultured hippocampal neurons to determine the underlying mechanisms for the anticonvulsant properties of SSa.

## Materials and Methods

### 1. Reagents

Neurobasal-A medium, B27, Fetal Bovine Serum and Horse Serum were obtained from Gibco-BRL (Invitrogen Corp., San Diego, CA). Saikosaponin a (purity >98%, obtained from Shanghai Institute of Pharmaceutical Industry, Shanghai, China) was dissolved at a concentration of 100 mM in DMSO as a stock solution and stored at −20°C. Working solutions were prepared before each experiment, and the final DMSO concentration did not exceed 0.1% throughout the study. Recordings were performed in the presence and absence of SSa or the vehicle. Other reagents were purchased from Sigma (St. Louis, MO, USA).

### 2. Ethics Statement

All animals were purchased from the Animal Center of the Fourth Military Medical University. The animals were housed and handled in strict accordance with the guidelines of the institutional and national Committees of Animal Use and Protection. The protocol was approved by the Committee on the Ethics of Animal Experiments of the Fourth Military Medical University (Permit Number: SCXK 2007-007).

**Figure 3:**
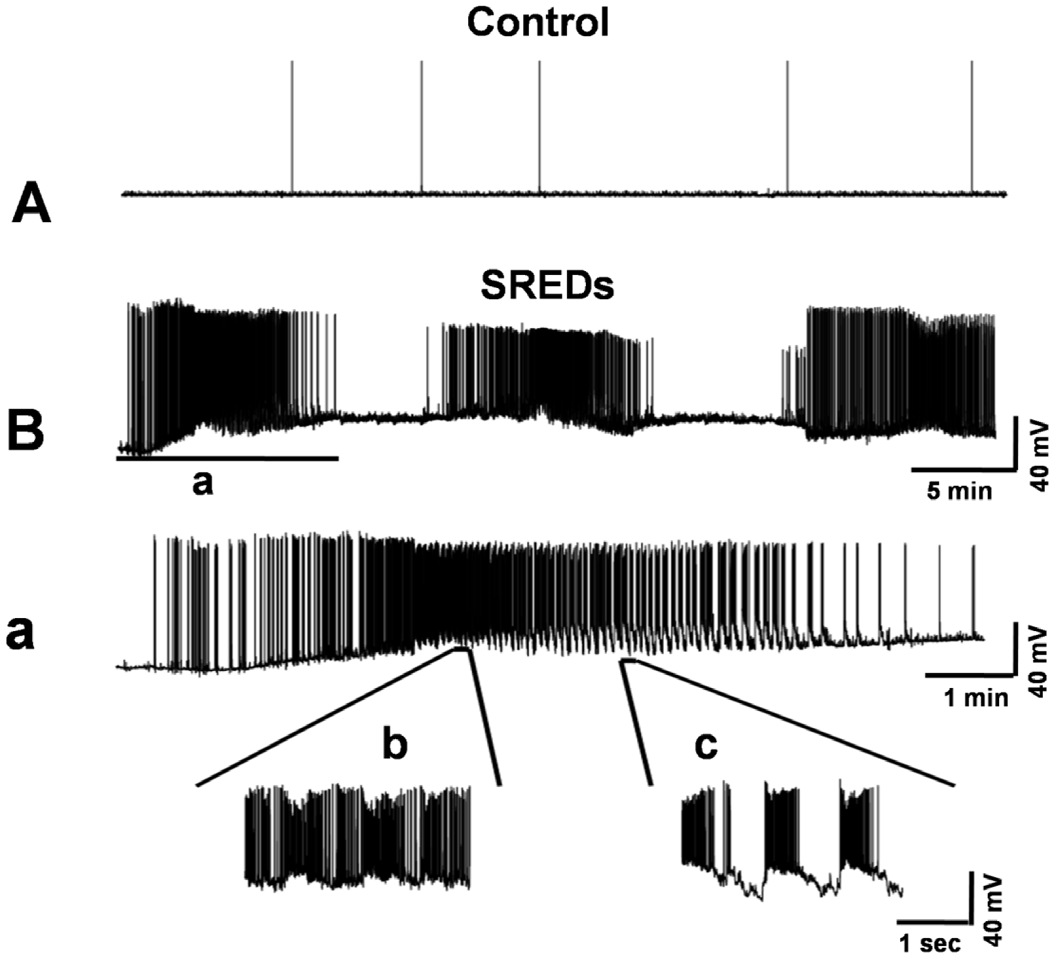
Induction of SREDs in cultured hippocampal neurons following a 3 h exposure to low-Mg^2+^ solution. Whole-cell current-clamp recordings were obtained from cultured hippocampal neurons. (A) A representative recording from a control neuron displays intermittent spontaneous action potentials that are consistently observed during basal activity in this hippocampal culture preparation. (B) A continuous 45 min recording from a representative neuron 24 h following exposure to 3 h of low-Mg^2+^ solution. The pathophysiological state of SREDs in this in vitro preparation is evident by the presence of three independent spontaneous seizure episodes. (a) An expansion of the corresponding segment in the trace above (B) to discriminate the high-frequency burst discharges that overlie individual PDSs, a pathophysiological characteristic observed in epilepsy, and the numerous spikes associated with each SRED. (b) and (c) further expansions of two segments from (a) display two types of PDSs with spike frequency >3 Hz.

**Figure 4:**
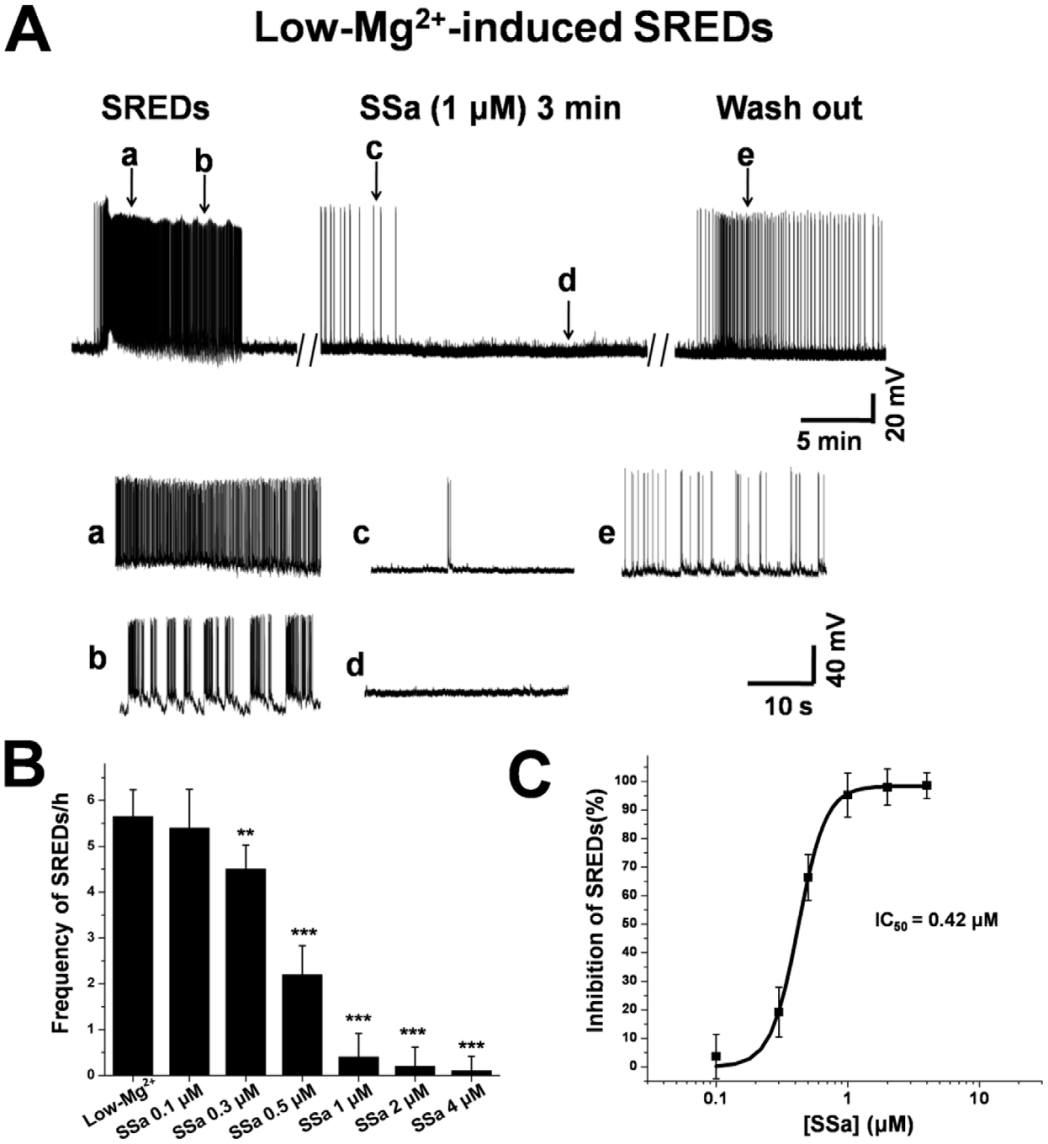
SSa inhibits low-Mg^2+^-induced SREDs activity in cultured hippocampal neurons. (A) A representative whole-cell current-clamp recording from a neuron during pBRS (with vehicle) displaying SREDs activity (a, b), after application of SSa (1 µM; c, d), and during wash out with pBRS (e). Inserted segments (a–e, lower panel) were expansions of the original traces at the arrows indicated and showed details of the inhibitory effect on SREDs activity. (B) Bar graph representing average frequencies of SREDs in the absence (pBRS with vehicle) and presence of SSa (0.1 µM to 4 µM; n = 11; paired t-test; **P<0.01, ***P<0.001 vs. pBRS). (C) Log concentration-response curve for SSa on the percentage inhibition of SREDs was determined by least-square linear regression analysis (n = 11; *IC_50_* = 0.42 µM).

### 3. Hippocampal Neuronal Culture

Studies were conducted on primary mixed cultured hippocampal neurons which were prepared as previously described with a slight modifications [4]. In brief, hippocampal cells were prepared from 1–2 d postnatal Sprague-Dawley rats and plated at a density of 2.5×10^4^ cells/cm^2^ onto a glial support layer that was previously plated onto poly-L-lysine–coated (0.05 mg/ml) glass coverslips. Cultures were maintained at 37°C in a 5% CO_2_/95% air atmosphere and half of the medium was replaced twice weekly with Neurobasal-A medium supplemented with 2% B-27, 0.5 mM L-glutamine, 100 units/ml penicillin, 100 g/ml streptomycin and 0.25 mg/ml fungizone.

### 4. Induction of SREDs and SE by Low-Mg^2+^ Treatment of Cultured Hippocampal Neurons

After 2 weeks, cultures were utilized for experimentation. A coverslip with cultured neurons was placed in a recording chamber and constantly superfused with physiological bath recording solution (pBRS) with or without MgCl_2_ (1 mM) containing (in mM): 145 NaCl, 2.5 KCl, 10 HEPES, 2 CaCl_2_, 10 glucose and 0.002 glycine, pH 7.3, with the osmolarity adjusted to 325±5 mOsm with sucrose [22]. Thus, low-Mg^2+^ treatment was carried out with pBRS without added MgCl_2_, whereas sham controls were treated with pBRS containing 1 mM MgCl_2_. Unless indicated as low-Mg^2+^ treatment, experimental protocols in this study utilized pBRS containing 1 mM MgCl_2_.

SE was induced by exposing cultured hippocampal neurons to pBRS without MgCl_2_ (low-Mg^2+^). Briefly, after removal of maintenance media, cultures were washed gently with 3×1.5 ml of Low-Mg^2+^ solution and then allowed to incubate in the Low-Mg^2+^ solution at 37°C under 5% CO_2_/95% air. The SE continued until pBRS containing 1 mM MgCl_2_ was added back to the cultures.

A 24 h maintenance culturing following 3 h exposure of low-Mg^2+^ solution to cultured hippocampal neurons was employed to induce SREDs using established procedures [22]. It is the 3 h of low-Mg^2+^-induced SE injury that results in plasticity changes that underlie the SRED activity [4]. In brief, at the end of treatment the cultured hippocampal neurons with low-Mg^2+^ solution for 3 h, the cultures were washed gently with 3×1.5 ml of Neurobasal-A medium at 37°C, and then incubated in the Neurobasal-A medium at 37°C under 5% CO_2_/95% air for 24 h.

**Figure 5:**
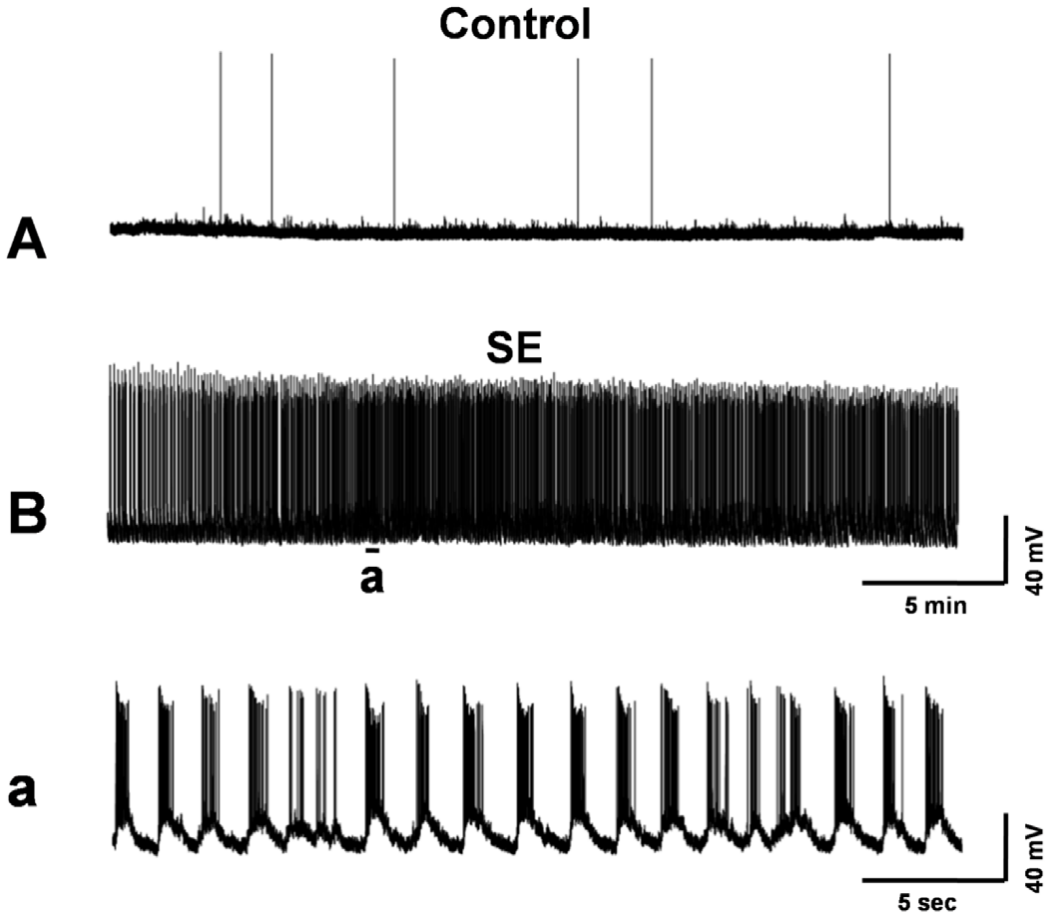
Induction of SE in cultured hippocampal neurons during exposure to low-Mg^2+^ solution. Continuous whole-cell current-clamp recordings were obtained from hippocampal neurons before and after exposure to low-Mg^2+^ solution. (A) A representative recording from a control neuron showed occasional spontaneous action potentials. (B) A representative recording during low-Mg^2+^ treatment showed continuous tonic high-frequency epileptiform bursts (SE), with spike frequencies ranging from 5 Hz to 18 Hz. (a) An expansion of a segment from (B) showed the presence of continuous burst discharges, revealing the individual epileptiform bursts, each consisting of depolarization shifts overlaid with multiple spike activity.

**Figure 6:**
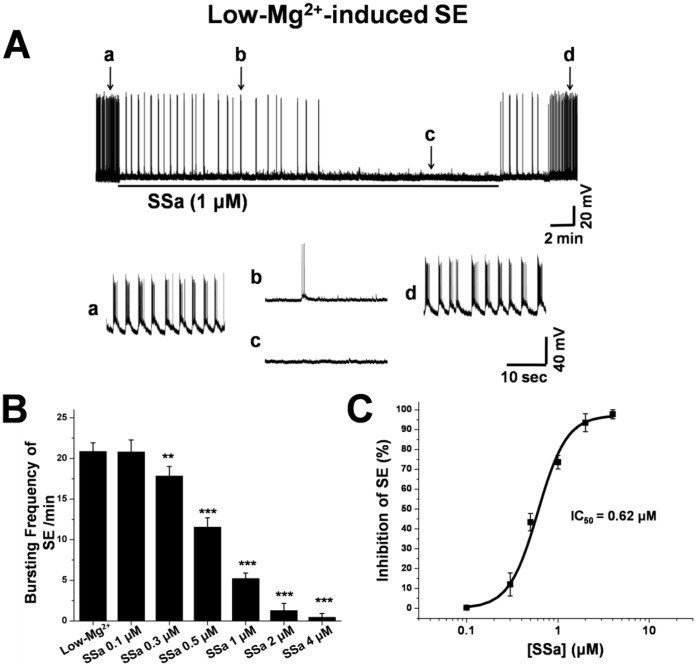
SSa inhibits low-Mg^2+^-induced SE activity in cultured hippocampal neurons. (A) A representative recording from a cultured hippocampal neuron exposure to low-Mg^2+^ solution. SE activity (with vehicle; a) was significantly inhibited in the presence of SSa (1 µM; b, c) and restored after wash out for 8–10 min (d). Inserted segments (a–d, lower panel) were expansions of the original traces at the times indicated and showed details of the inhibitory effect on SE activity. (B) Bar graph representing average frequencies of SE in the absence (low-Mg^2+^ solution with vehicle) and presence of multiple concentrations of SSa (0.1 µM to 4 µM; n = 10; paired t-test; **P<0.01, ***P<0.001 vs. Low-Mg^2+^). (C) The log concentration-response curve was determined for SSa by determining percentage inhibition of SE frequency after the application of multiple concentrations of SSa with an *IC_50_* = 0.62 µM (n = 11).

### 5. Electrophysiological Recordings

Individual neurons were visualized with a 40X water-immersion objective under a microscope (Olympus, Japan) equipped with infrared differential interference contrast optics. Whole-cell current and voltage-clamp recordings were carried out using an Axopatch 200B amplifier (Axon Instruments, Foster City, CA). The data were sampled at 2 kHz for current-clamp recordings and 20 kHz for voltage-clamp recordings. The data were transferred to a PC using a Digidata 1322A (Axon Instruments) interface and acquired using pCLAMP 9 (Axon Instruments) software. Patch electrodes with a resistance of 2–4 MΩ in the bath were pulled on a microelectrode puller (P97, Sutter Instruments, USA). After small negative pressure formed a gigaohm seal, whole-cell recordings were established by rupture of the cell membrane with further negative pressure. Only neurons with resting membrane potentials ≤ −50 mV were selected for further study. The junction potentials were 17.2 mV for current-clamp recordings, 9.8 mV for NMDA current recordings and 3.7 mV for sodium recordings and were corrected accordingly. Recordings were terminated if the access resistances (Ra) increased or the resting membrane potentials dropped by 20% or more from control levels. For voltage-clamp recordings, fast and slow capacitances were neutralized and Ra (4–10 MΩ) was compensated (70–90%) and periodically monitored. The cell membrane potential was held at −60 mV. Current-clamp recordings were performed at 32–33°C, while voltage-clamp recordings were performed at 24–25°C.

#### 5.1 Whole-cell current-clamp recordings in low-Mg^2+^ treated cultured hippocampal neurons

Whole-cell current-clamp recordings were performed using previously established procedures. For SE studies, coverslips with cultured neurons were placed in a recording chamber and superfused with low-Mg^2+^ solution to induce high-frequency burst discharges. For analysis of SREDs, cultures were previously exposed to low-Mg^2+^ solution for 3 hours, and then the neurons were returned to a maintenance medium for 24 h, after which the cultured neurons were superfused with pBRS during recordings. Electrodes were filled with an internal solution containing (in mM): 140 K-gluconate, 1 MgCl_2_ and 10 HEPES, pH 7.2 with KOH. Different concentrations of SSa or DMSO were included in the recording solution and applied to the recording chamber via the perfusion system (Warner Instruments, Hamden, CT). Drugs were applied only after the membrane potentials and population spikes were stabilized.

#### 5.2 Whole-cell voltage-clamp recordings of NMDA-evoked current, *I_NaP_* and *I_Nat_* in cultured hippocampal neurons

The 10–14 d cultured hippocampal neurons were used for voltage-clamp recordings. Standard voltage-clamp techniques were performed using previously established procedures. To record NMDA-induced current [23], the coverslip with cultured neurons was placed in a recording chamber and constantly superfused with pBRS, at which time 1 µM TTX, 20 µM CNQX and 20 µM bicuculline was added. Electrodes were filled with an intracellular solution containing (in mM): 120 K-gluconate, 10 KCl, 5 NaCl, 1 CaCl_2_, 2 MgCl_2_, 11 EGTA, 10 HEPES, 2 Mg-ATP and 0.1 Na-GTP, pH 7.3 with KOH. Rapid local drug application to the cultured hippocampal neurons was performed using a ‘Y-tube’ perfusion system, and the application capillaries were positioned a few hundred micrometers from the cell under study [23]. When recording NMDA current,100 µM NMDA, with 1 µM TTX, 20 µM CNQX and 20 µM bicuculline in Mg^2+^-free pBRS, was applied locally for 30 s every 5 min to minimize desensitization-induced decrease of current amplitude at a holding potential of −60 mV. For SSa treatment conditions, different concentrations of SSa or DMSO were delivered with NMDA simultaneously.

As for *I_NaP_ and I_Nat_*, the external solution contained (in mM): 138 NaCl, 10 HEPES, 2.5 KCl, 2 CaCl_2_, 1 MgCl_2_,10 glucose, 10 TEA and 4 4-AP (blockers of K^+^ channels) and 50 µM CdCl_2_ (non-selective blocker of Ca^2+^ channels), pH 7.3 with NaOH. The CsCl-based internal solution contained the following components (in mM): 140 CsCl, 10 NaCl, 2 MgCl_2_, 10 EGTA, 10 HEPES, 2 Mg-ATP and 0.1 Na-GTP, pH 7.2 with CsOH [24].

**Figure 7:**
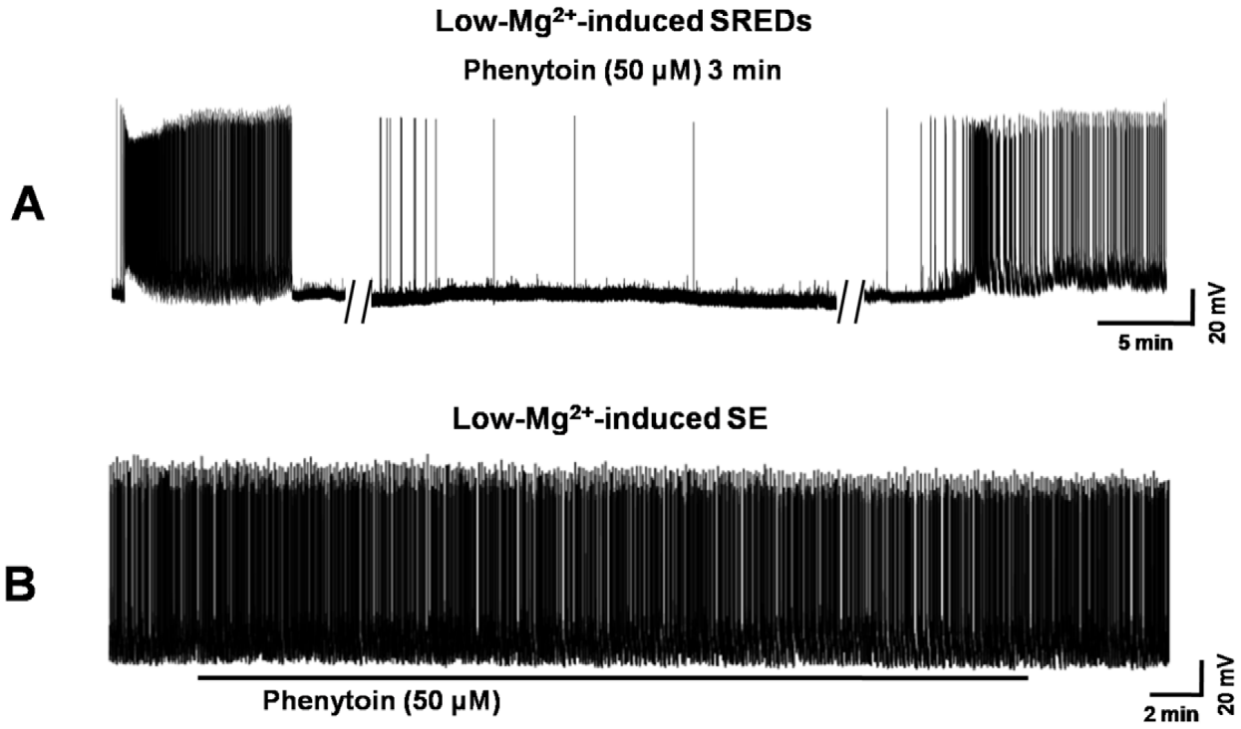
Evaluation of anticonvulsant effects of Phenytoin on low-Mg^2+^-induced SREDs and SE activities. (A) Application of Phenytoin (50 µM) effectively suppressed SREDs activity. (B) Application of Phenytoin (50 µM) had no inhibitory effect on SE activity.

**Figure 8:**
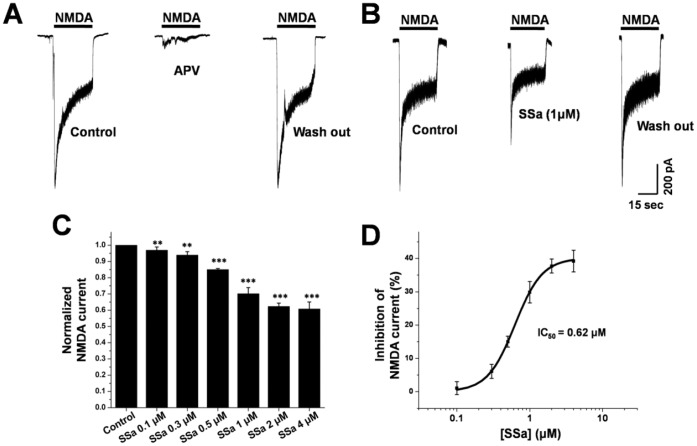
SSa inhibits NMDA-evoked current in cultured hippocampal neurons. (A) After the application of NMDA (100 µM), a robust inward current was produced (first) which was completely eliminated by APV (50 µM) (middle). (B) Representative current traces from a cultured hippocampal neuron before and after the application of SSa (1 µM). NMDA-evoked current was reversibly inhibited by the application of SSa (1 µM). (C) Bar graph showing significant inhibition of peak NMDA-evoked current before (control with vehicle) and after the application of SSa (n = 10; paired t-test; **P<0.01, ***P<0.001 vs. control). (D) Log concentration-response curve for the percentage inhibition of SSa on peak NMDA-evoked current (*IC_50_* = 0.62 µM).

### 6. Data Analysis

All results were expressed as the mean ± SEM. Statistical analysis was performed using Statistical Product and Service Solutions (SPSS) software (paired t-test, unpaired t-test or one-way AVOVA). Values of *P*<0.05 were considered to be significant. The data were plotted using Origin 8.0.

SE frequency was determined by counting individual epileptiform bursts over a recording duration of 30 min for each neuron analyzed, while SREDs frequency was determined by counting individual SREDs over a recording duration of 60 min for each neuron analyzed. For both SE and SREDs, the percentage inhibition of frequency before and after applying SSa was determined and the statistic analysis was carried out on multiple hippocampal neurons at each concentration of SSa. For NMDA-evoked current and sodium currents, the normalized current and the percentage inhibition of peak amplitude were determined and statistical analysis was carried out on multiple hippocampal neurons at each concentration of SSa.

For concentration-response analysis, the percentage inhibition of frequency of SREDs or SE, and the percentage inhibition of peak amplitude of NMDA-evoked current or sodium currents were determined at different concentrations of SSa. The concentration-response curve was obtained by fitting the experimental data with a logistic equation with variable slope (Hill coefficient): *y/y_max_* = {1-[*D/*(*D*+*IC_50_*)] ^n^}, where *y* is the response in the presence of drug, *y_max_* is the maximal response in the absence of drug, *D* is the drug concentration, *IC_50_* is the concentration of drug producing a half maximal inhibition of the response and *n* is the Hill coefficient. Fit estimates were calculated using Levenberg-Marquardt least squares ALGORITHM.

**Figure 9:**
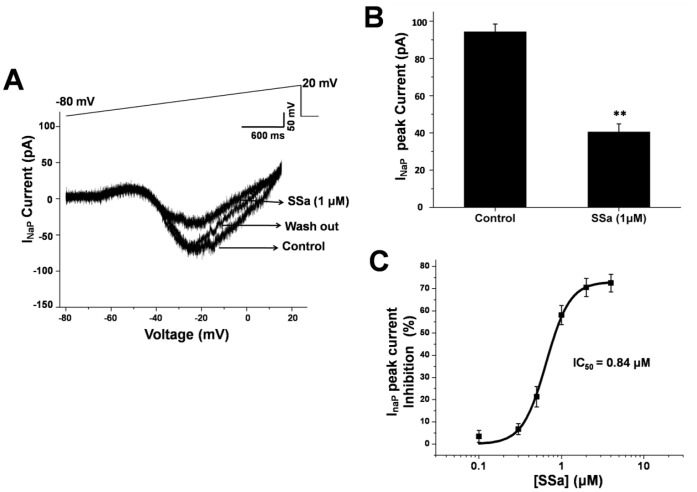
SSa inhibits the peak of *I_NaP_* in cultured hippocampal neurons. (A) Traces of *I_NaP_* (bottom) induced by slow depolarizing voltage-ramps (from −80 mV to +20 mV, top) in a cultured hippocampal neuron in control (with vehicle), the presence of SSa (1 µM) and washing out conditions. (B) Bar graph showing that average peak *I_NaP_* in the presence of SSa (1 µM) was significantly decreased in cultured hippocampal neurons (n = 8; paired t-test; **P<0.01 vs. control). (C) Log concentration-response curve showing the percentage inhibition of SSa on peak *I_NaP_* (*IC_50_* = 0.84 µM).

**Figure 10:**
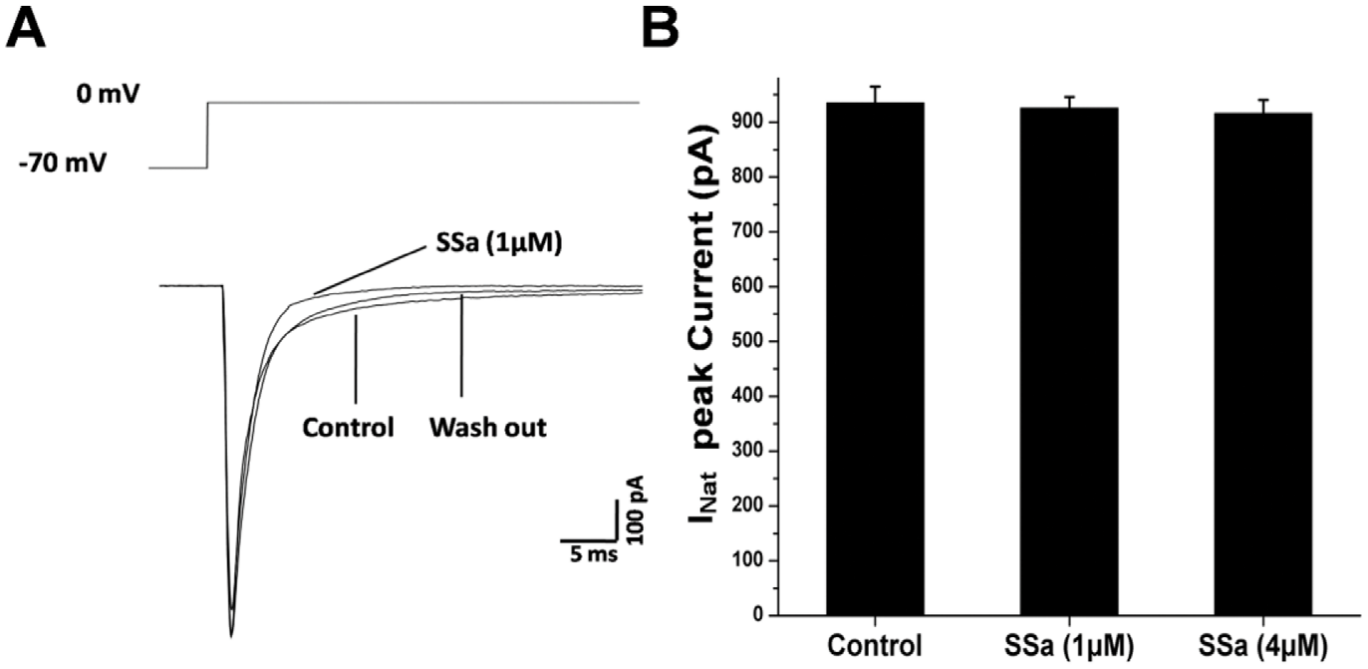
SSa has no significant effect on *I_Nat_* in HNCs. (A) Traces of *I_Nat_* (bottom) induced by a 30 ms voltage step (from −70 mV to 0 mV, top) injected to a cultured hippocampal neuron in control (with vehicle), the presence of SSa (1 µM and 4 µM) and washing out conditions. There were no visible changes after applying SSa. (B) Bar graph showing that SSa (1 µM and 4 µM) had no significant inhibition on the peak *I_Nat_* in cultured hippocampal neurons (n = 8; paired t-test; *P*>0.05).

## Results

### Effects of SSa on Membrane Properties and Excitability of Cultured Hippocampal Neurons

Under the conditions with Kynurenic acid (2 mM) and picrotoxin (0.1 mM) added to the external solution to block fast synaptic transmission, we examined effects of SSa on the basic membrane properties including the resting membrane potential and the input resistance (R_in_). SSa did not produce any significant effect on resting membrane potential (−61.2±0.5 mV of control vs. −60.9±0.6 mV of SSa at 1 µM; n = 10, paired t-test; *P*>0.05; Table 1). R_in_, was tested by small currents (−30 pA to 30 pA, 400 ms) injection at a holding potential of −60 mV. There was no significant effect on R_in_ after applying SSa (165.3±12.1 MΩ of control vs. 168.5±8.5 MΩ of SSa at 1 µM; n = 10, paired t-test; *P*>0.05; Table 1). Excitability was measured as the number of action potentials induced by a depolarizing current (150 pA, 600 ms) injection at a holding potential of −60 mV. The average number of spikes elicited decreased from 17.5±0.3 of the control to 13.2±1.6 after applying SSa (1 µM; n = 10; paired t-test; *P*<0.01; Fig. 2A–2B). These data suggest that SSa could inhibit the excitability of hippocampal neurons.

### Effects of SSa on SREDs and SE in the HNC Models of AE and SE

To evaluate the anticonvulsant activities of SSa, whole-cell current-clamp recordings were carried on the HNC models of AE and SE. The anticonvulsant effects of SSa were observed with concentrations as low as 0.1 µM and at a maximum of 4 µM. The frequency and the percentage inhibition of SREDs or SE were determined (see Materials and Methods) to evaluate the effectiveness of SSa.

To induce SREDs, 2-week-old cultured hippocampal neurons were exposed to low-Mg^2+^ solution for 3 h and then returned to a maintenance medium for 24 h. Recordings from control neurons displayed intermittent spontaneous action potentials that were consistently observed during basal activity in the hippocampal culture preparations (Fig. 3A). While, 24 h following exposure to low-Mg^2+^ solution, whole-cell current-clamp recordings in pBRS showed a permanent plasticity change evidenced by the presence of SREDs (Fig. 3B). Each SRED was comprised of multiple paroxysmal depolarization shifts (PDSs) that were overlaid with poly spikes (Fig. 3a–3c). The presence of PDSs is a pathophysiological characteristic similar to what is observed in clinical epilepsy [25].

SSa was applied to the neurons only after the SREDs were stable. Application of SSa (1 µM) for 5–8 min almost fully inhibited the expression of SREDs (Fig. 4A, 4c–4d). Then, another 60 min was taken to observe the stable anticonvulsant activities of the drug against SREDs. The inhibition of SSa on SREDs was reversible after being washed out with pBRS for 5–10 min (Fig. 4A, 4e). The frequency of SREDs per hour before and after applying SSa and the percentage inhibition of SREDs were measured. SSa significantly inhibited the frequency of SREDs per hour from 5.7±0.6 (n = 11) to 4.5±0.5 (n = 11) at concentration as low as 0.3 µM (paired t-test; *P*<0.01; Fig. 4B), at which concentration the percentage inhibition of SREDs was 19.2±8.7%. SSa inhibited SREDs in a concentration-dependent manner with an *IC_50_* = 0.42 µM (Fig. 4C).

We further evaluated the anticonvulsant properties of SSa in the HNC model of SE. Whole-cell current-clamp recordings from 2-week-old control neurons showed baseline activity with the occasionally spontaneous action potentials (Fig. 5A). Removal of MgCl_2_ (low-Mg^2+^) from the recording solution resulted in SE (Fig. 5B). This hyperexcitable state consisted of repetitive individual burst discharges (Fig. 5a), and each burst was comprised of multiple spikes that overlay a depolarization shift. The continuous epileptiform activity in this HNC model of SE is characterized by the spike frequency >3 Hz and lasting more than 30 min during the low-Mg^2+^ exposure [26].

Compared with low-Mg^2+^ treatment alone (Fig. 6A, 6a), SSa (1 µM) completely inhibited SE after being applied for 10–15 min (Fig. 6A, 6b–6c). After washing out the drug with low-Mg^2+^ solution for 5–8 min, SE activity returned (Fig. 6A, 6d). The frequency of epileptiform burst discharges per minute (20.9±1.1; n = 10) was significantly decreased by SSa at concentration as low as 0.3 µM (17.8±1.2; n = 10; paired t-test; *P*<0.01; Fig. 6B). The log concentration-response curve of percentage inhibition of SE showed that SSa inhibited SE in a concentration-dependent manner and that the percentage inhibition of SE by SSa at 0.3 µM, 0.5 µM, 1 µM, 2 µM and 4 µM was 12.1±5.8%, 43.4±4.4%, 73.6±3.4%, 93.5±4.5% and 97.8±2.3%, respectively (n = 10; Fig. 6C). The *IC_50_* was 0.62 µM.

Comparison of anticonvulsant effects against SREDs and SE of SSa with another AED, Phenytoin. Phenytoin (50 µM), fully blocked SREDs (Fig. 7A) in the HNC model of AE, while having no effect on reducing low-Mg^2+^-induced SE in the HNC model of SE (Fig. 7B).

### Effects of SSa on NMDA-evoked Current in Cultured Hippocampal Neurons

It was reported that both low-Mg^2+^-induced SREDs and SE induced cell death were dependent on a NMDA receptor/Ca^2+^ transduction pathway [27], [28], implying the potential change of NMDA receptor current within the SREDs and SE. In this study, SSa could fully inhibit SREDs and SE (see above), so we further evaluated the effects of SSa on NMDA-evoked current by using whole-cell voltage-clamp recordings. After local puff of NMDA (100 µM) for 30 s, a robust inward current was evoked (Fig. 8A, first), which could be completely inhibited by APV (50 µM; Fig. 8A, middle). Application of SSa (1 µM) caused an evident and reversible reduction in the NMDA-evoked current in cultured hippocampal neurons (Fig. 8B). Normalizing the peak NMDA-evoked current, SSa significantly decreased the NMDA-evoked current peak amplitude at every concentration (n = 10; paired t-test; *P*<0.01 vs. control; Fig. 8C). SSa inhibited the peak NMDA-evoked current in a concentration-dependent manner with an *IC_50_* = 0.62 µM, and the percentage inhibition of the peak NMDA-evoked current was 1.07±2.0%; 6.11±2.1%; 15.01±1.6%; 29.87±3.2%; 37.73±2.1% and 39.22±3.3% at 0.1 µM; 0.3 µM, 0.5 µM, 1 µM, 2 µM and 4 µM, respectively (n = 10; Fig. 8D).

### Effects of SSa on *I_NaP_* and *I_Nat_* in Cultured Hippocampal Neurons

In this study, SSa significantly inhibited both elicited action potentials in cultured hippocampal neurons and repetitive (burst) firing in the HNC models of AE and SE (Fig. 2A; Fig. 4A and Fig. 6A). We further evaluated effects of SSa in cultured hippocampal neurons on sodium currents, including *I_NaP_* and *I_Nat_*, which are critical for action potentials and burst firing. Using whole-cell voltage-clamp recordings, *I_NaP_* was evoked by slow depolarizing voltage-ramps from −80 mV to +20 mV, which inactivated the large transient current [29]. SSa reversibly suppressed *I_NaP_* (Fig. 9A). The average current peak of *I_NaP_* in cultured hippocampal neurons was 94.3±4.2 pA (control; n = 8). SSa (1 µM) significantly inhibited the peak amplitude of *I_NaP_* (40.5±4.4 pA; n = 8; paired t-test; *P*<0.01 vs. control; Fig. 9B). SSa significantly inhibited the peak amplitude of *I_NaP_* by 21.3±4.6% at concentration as low as 0.5 µM (n = 8), and the inhibitory effects were in a dose-dependent manner with an *IC_50_* = 0.84 µM (n = 8; Fig. 9C).

A 30 ms depolarization voltage step (from −70 mV to 0 mV) was used to evoke *I_Nat_* with whole-cell voltage-clamp recordings [24]. As shown in Fig. 10A, SSa (1 µM) almost had no effect on the *I_Nat_* peak amplitude. Even at the highest concentration of 4 µM, SSa had no significant reduction on the peak *I_Nat_* (n = 8; paired t-test; *P*>0.05; Fig. 10B). These results suggest that SSa might have a selective effect on *I_NaP_*.

## Discussion

The primary finding of the current study is that SSa acts as an anticonvulsant in a concentration-dependent manner against low-Mg^2+^-induced SREDs and SE in the HNC models of AE and SE. SSa inhibited NMDA-evoked current and *I_NaP_* in cultured hippocampal neurons, which may imply the potential antiepileptic mechanisms of SSa.

### The HNC Models of AE and SE

The HNC models of AE and SE used in this study simulate many electrophysiological features, for example that in zero external magnesium medium, pyramidal neurons from high-density cultures produced recurrent spontaneous action potential bursts superimposed on prolonged depolarizations, which were partially attenuated by the NMDA receptor antagonist APV [5], [6]. The models are well suited to carry out biochemical, molecular [30] and electrophysiological investigations [4]. In addition, the two in vitro models have been commonly used to study the mechanisms underlying seizures, seizure-induced plasticity, physiology and molecular changes and development of pharmacoresistant seizures [31], [32].

SE was induced after exposing cultured hippocampal neurons to low-Mg^2+^ solution, with spike frequency within epileptiform bursts ranging from 5 Hz to 18 Hz. We used neurons 24 h after 3 h exposure to low-Mg^2+^ solution in this HNC model of AE, in which condition hippocampal neurons develop SREDs with high-frequency spike firing >3 Hz [25]. The two in vitro models are amenable to experimental manipulation, allow for direct analysis of neurons undergoing SREDs and SE in vitro and are valuable tools for the pharmacological studies on the anticonvulsant effects of various investigational compounds [22], [33].

During exposure of epileptic cultures with SREDs or SE to SSa, we found that SSa had anticonvulsant effects manifested by a decrease in frequencies of SREDs and SE in a concentration dependent manner, with an *IC_50_* of 0.42 µM and 0.62 µM, respectively. SSa displayed full suppression of SREDs at 1 µM which is as efficacious as phenytoin (50 µM). SSa also produced a full suppression of SE at a higher concentration of 4 µM, while, phenytoin and phenobarbital which are used as clinical anticonvulsants could not abolish low Mg^2+^-induced SE, even at very high concentrations up to 150 µM [22], [33]. As we know, the higher dose of the AEDs, the more adverse effects in clinical treatment of epilepsy. The fact that SSa inhibited SREDs and SE in a small dose may have clinical implications for considering the use of SSa in the treatment of epilepsy. On the other hand, SE is often refractory to conventional anticonvulsant treatments, and some conventional AEDs, such as Phenobarbital [22], Ethosuximide and Carisbamate [33], were not able to abolish SE. The ability of SSa to abolish SE in a small dose in vitro is an important evidence that SSa may have significant clinical implications for the treatment of refractory epilepsy.

### NMDA Receptor Current in SREDs and SE

Mg^2+^ is a blocker of the ionotropic NMDA receptor channel [7]. Exposure to a low-Mg^2+^ solution will result in repetitive depolarizations and excessive transmitter release presynaptically and activation of NMDA receptor postsynaptically, which would increase neuronal network excitability in vitro SREDs and SE [6], [8]. It was reported that low-Mg^2+^-induced SREDs were dependent on an NMDA receptor/Ca^2+^ transduction pathway in the HNC model of AE [27]. A sustained elevation of intracellular Ca^2+^ concentration gated through the NMDA receptor channel is required for the development of SREDs [27]. Although previous reports have shown that SE was not significantly inhibited by blocking NMDA receptors [34], the SE-mediated activation of NMDA receptors induced significant cell death, and the neuronal death was reduced by NMDA receptor channel inhibition [34]. In this study, we demonstrated that SREDs and SE in the HNC models of AE and SE could be fully inhibited by SSa. Thus, we further evaluated the effect of SSa on NMDA receptor current. Our study demonstrated that exogenous application of NMDA (100 µM) evoked a robust inward current, which was completely eliminated by APV (50 µM), a NMDA receptor antagonist. SSa significantly decreased the peak amplitude of the NMDA-evoked current in a concentration-dependent manner.

The NMDA receptor is one of the ionotropic glutamate receptors that mediate excitatory neuronal transmission. Activation of the NMDA receptor results in the opening of a non-selective cation channel, which allows flow of Na^+^ and Ca^2+^ ions into the cell. Alterations in Ca^2+^ homeostasis through NMDA receptor activation have been observed in a number of models of neuronal excitotoxicity [35] and pathophysiology, including AE [36] and SE [37]. Our data suggest that decreasing NMDA receptor current may be a potential anticonvulsant mechanism of SSa.

### Sodium Current in Anticonvulsant Mechanism

Sodium channels control the rising phase and the propagation of the action potential and determine the duration and frequency of repetitive neuronal firing of neurons. Previous studies have reported that the abnormal function of sodium channels increases the excitability of neurons and contributes to the generation and spread of abnormal discharges and epileptic seizures [11]. The inhibition of neuronal sodium currents is commonly accepted as a prominent antiepileptic mechanism [12], [13]. Several established AEDs, including phenytoin [38] and carbamazepine [39], as well as the newer AEDs, lamotrigine [40] and topiramate [41], have been shown to suppress abnormal neuronal excitability by inhibiting sodium currents in cultured rat central neurons.

Our whole-cell current-clamp data showed that SSa could inhibit both elicited action potentials and repetitive (burst) firing. We further examined whether SSa also had effects on sodium channels, which play an important role in action potentials and repetitive firing. We evaluated modulations of SSa on two types of sodium currents, *I_NaP_* and *I_Nat_*. Our study demonstrated that although *I_NaP_* activation curves were not significantly different from those of the control groups after the application of SSa, the peak amplitude of *I_NaP_* was significantly decreased in cultured hippocampal neurons. Further study on *I_Nat_* showed that SSa had no significant inhibition of the peak amplitude of *I_Nat_* in cultured hippocampal neurons.

*I_NaP_*_,_ a non-inactivating component of the total sodium currents that persists after the rapid decay of *I_Nat_*_,_ can facilitate repetitive firing [10]. After activation by a small synaptic depolarization, it will augment the membrane potential closer to firing threshold, and promote high-frequency firing, thereby lowering the threshold for epileptic activity [10], [42]. Several studies have provided evidence that limbic seizures induce an increase in *I_NaP_* [43]. In addition, PDSs were hypothesized to be sustained by *I_NaP_*. The blockade of *I_NaP_* by different AEDs has been reported, such as phenytoin, valproic acid, lamotrigine, and topiramate [44], [45], [46], [47]. These results suggest that decreasing *I_NaP_* may contribute to the anticonvulsant properties of SSa.

In conclusion, the results of this study show, for the first time, that SSa has anticonvulsant properties in HNC models of AE and SE. The effective suppression of the epileptiform discharge of both SREDs and SE in vitro by SSa may have a clinical implication in the treatment of epilepsy. Furthermore, the inhibitions of both the NMDA-receptor current and *I_NaP_* in cultured hippocampal neurons may imply the potential anticonvulsant mechanism of the drug. Because there were little studies on the changes of NMDA-receptor current or sodium channel currents within the HNC models of AE and SE, in this study we evaluated the effects of SSa on NMDA-receptor current and sodium channel currents in normal cultured hippocampal neurons, which may imply the potential pathomechanism. Further studies in epilepsy will be designed to explore the anticonvulsant mechanism of SSa.
